# RNA-Binding Domain in the Nucleocapsid Protein of Gill-Associated Nidovirus of Penaeid Shrimp

**DOI:** 10.1371/journal.pone.0022156

**Published:** 2011-08-03

**Authors:** Chumporn Soowannayan, Jeff A. Cowley, Wojtek P. Michalski, Peter J. Walker

**Affiliations:** 1 CSIRO Livestock Industries, Queensland Bioscience Precinct, St. Lucia, Queensland, Australia; 2 CSIRO Livestock Industries, Australian Animal Health Laboratory, Geelong, Victoria, Australia; 3 National Center for Genetic Engineering and Biotechnology (BIOTEC), National Science and Technology Development Agency, Thailand Science Park, Klong Luang, Patumthani, Thailand; 4 CENTEX SHRIMP, Faculty of Science, Mahidol University, Bangkok, Thailand; Queensland Institute of Medical Research, Australia

## Abstract

Gill-associated virus (GAV) infects *Penaeus monodon* shrimp and is the type species okavirus in the *Roniviridae*, the only invertebrate nidoviruses known currently. Electrophoretic mobility shift assays (EMSAs) using His_6_-tagged full-length and truncated proteins were employed to examine the nucleic acid binding properties of the GAV nucleocapsid (N) protein *in vitro*. The EMSAs showed full-length N protein to bind to all synthetic single-stranded (ss)RNAs tested independent of their sequence. The ssRNAs included (+) and (−) sense regions of the GAV genome as well as a (+) sense region of the M RNA segment of Mourilyan virus, a crustacean bunya-like virus. GAV N protein also bound to double-stranded (ds)RNAs prepared to GAV ORF1b gene regions and to bacteriophage M13 genomic ssDNA. EMSAs using the five N protein constructs with variable-length N-terminal and/or C-terminal truncations localized the RNA binding domain to a 50 amino acid (aa) N-terminal sequence spanning Met^11^ to Arg^60^. Similarly to other RNA binding proteins, the first 16 aa portion of this sequence was proline/arginine rich. To examine this domain in more detail, the 18 aa peptide (M^11^PVRRPLPPQPPRNARLI^29^) encompassing this sequence was synthesized and found to bind nucleic acids similarly to the full-length N protein in EMSAs. The data indicate a fundamental role for the GAV N protein proline/arginine-rich domain in nucleating genomic ssRNA to form nucleocapsids. Moreover, as the synthetic peptide formed higher-order complexes in the presence of RNA, the domain might also play some role in protein/protein interactions stabilizing the helical structure of GAV nucleocapsids.

## Introduction

Gill-associated virus (GAV) infects *Penaeus monodon* shrimp and is the type species of the genus *Okavirus* in the *Roniviridae*, the only currently known invertebrate nidoviruses. In all other nidoviruses, the nucleocapsid (N) protein is encoded by a gene located near to the genome 3′-terminus and downstream of genes encoding other virion structural proteins [1]. However, in GAV and other genotypic variants in the yellow head virus (YHV) complex, the N protein is encoded in the ORF2 gene which resides immediately downstream of the 20 kb 5′-terminal ORF1a/1b replicase gene [2], [3]. The deduced molecular masses of the 144–146 amino acid (aa) N proteins of GAV and YHV (16.0–16.3 kDa) are lower than those estimated by SDS-PAGE (20–22 kDa), which for YHV has been reported to be due to a C-terminal cluster of acidic residues [3], [4]. Immuno-electron microscopy has confirmed that the N protein is the primary structural protein component of okavirus nucleocapsids [2], [5].

Amongst strains of genotypes 1 (YHV), 2 (GAV), 3, 4 and 5 in the YHV complex [6], most amino acid variations (up to 17.2%) in the deduced N protein sequence occur in the highly charged N- and C-terminal domains [3], [6]. Nonetheless, the N proteins of GAV and YHV share common antigenic sites as evidenced by their cross-reactivity for a YHV N protein monoclonal antibody [5], [7] and polyclonal antiserum to a synthetic peptide designed to a C-terminal sequence of the GAV N protein [2]. As with the N proteins of coronaviruses and toroviruses [8], [9], the N proteins of GAV and YHV lack cysteine residues and are highly basic [2], [3]. It also possesses proline-rich and basic residue-rich domains likely to facilitate RNA binding as hypothesized for similar sequences in the N protein of toroviruses [2], [10]. The N protein length in okaviruses is intermediate to the corresponding proteins of arteriviruses (110–128 aa) and toroviruses (160–167 aa), and much shorter than the N protein of coronaviruses (377–454 aa) [2], [11], [12], [13], [14].

The process by which N proteins encapsidate genomic RNA to form nucleocapsids has been examined in many RNA viruses [8], [15], [16], [17] and in coronaviruses, as an example, the N protein interaction with RNA shows no preference for sequence, indicating that the specific nucleation of viral RNA likely requires additional factors [8], [18].

Here we have examined recombinant GAV N protein constructs in electrophoretic mobility shift assays (EMSAs) to identify its nucleic acid binding specificities *in vitro*, and its RNA binding domain, as initial steps to understanding the process by which nucleocapsids form in okaviruses. The N protein was found to bind ssRNA, dsRNA as well as ssDNA in a sequence independent manner and the RNA binding site was localized to an 18 aa proline/arginine-rich sequence near to its N-terminus.

## Results

### Expression and purification of recombinant GAV N protein constructs

**Figure 1:**
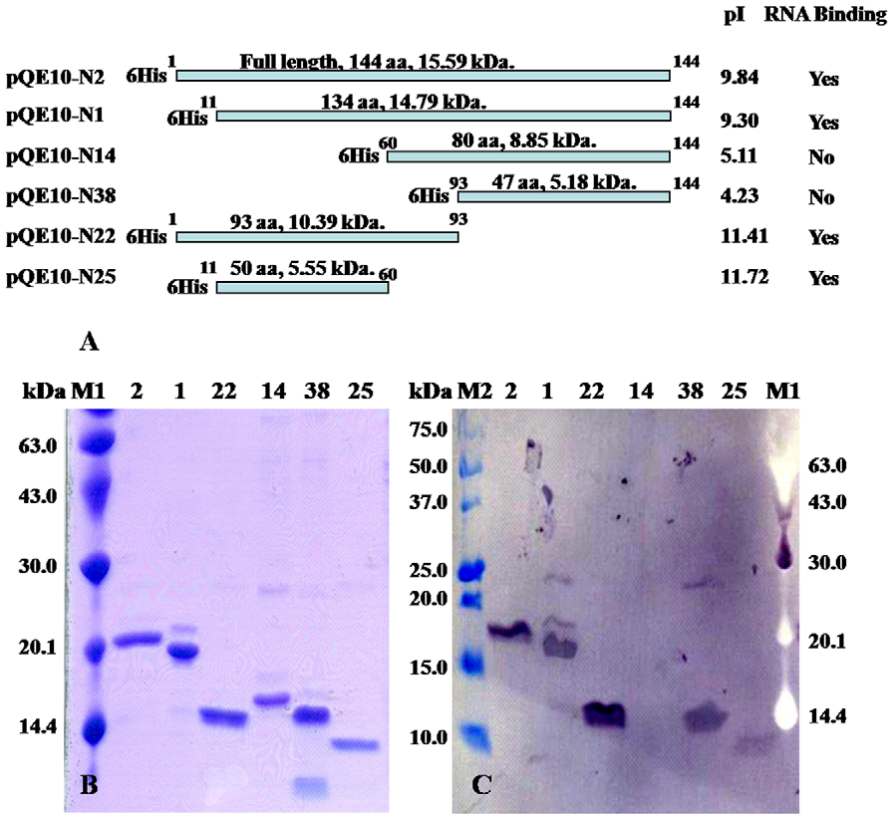
GAV N proteins used for electrophoretic mobility shift assay (EMSA). (A) Schematic representation of the His_6_-tagged recombinant GAV N protein (QE10-N2) and truncated N protein constructs (QE10-N1, -N14, -N38, -N22, -N25) used in EMSAs, including amino acid length, deduced molecular weight, pI and their ability to bind ssRNA. (B) CBB-stained SDS-PAGE gel showing the purity of Ni^2+^-purified His_6_-tagged fusion proteins. (C) Western blot of a duplicate gel with protein detected using Ni^2+^-HRP. M1  =  low range protein standards (Amersham Biosciences), M2  =  pre-stained Precision Plus All-Blue molecular weight standards (Bio-Rad).

A full-length ORF2 gene and five constructs designed to produce N proteins with N- and/or C-terminal truncations **(** **Fig. 1A** **)** were cloned into pQE10 and used to express recombinant His_6_-N fusion proteins in *E. coli*. The purity of the expressed proteins following Ni^2+^-NTA affinity chromatography and assessed by SDS-PAGE and CBB staining is shown in **Fig. 1B**. Migration of the full-length His_6_-N protein (QE10-N2; 144 aa, 16.0 kDa deduced mass) trailed the 20.1 kDa marker protein slightly, consistent with an elevated mass estimated previously [4] plus the additional ∼1 kDa mass of the N-terminal His_6_ sequence. The His_6_-N protein construct QE10-N1 (134 aa, 14.8 kDa) with a 10 aa N-terminal truncation migrated slightly below QE10-N2 and constructs QE10-N14 (84 aa, 9.3 kDa) and QE10-N38 (51 aa, 5.6 kDa) with 60 aa and 93 aa N-terminal truncations, respectively, migrated at lower masses of ∼16.5 and ∼15.5 kDa, respectively. Construct QE10-N22 (93 aa, 10.4 kDa) with a 51 aa C-terminal truncation migrated at ∼15 kDa, and protein QE10-N25 (50 aa, 5.52 kDa) with 10 aa and 60 aa N- and C-terminal truncations, respectively, migrated at ∼10 kDa.

In addition to using CBB-stained gels **(** **Fig. 1B** **)**, the fidelity of the recombinant His_6_-N protein constructs was confirmed in Western blots using horseradish peroxidase (HRP) labeled-Ni^2+^ to detect the His_6_-tag on each protein (**Fig. 1C**). Except for QE10-N14, all His_6_-tagged proteins were detected and were estimated to be >90% pure, with single bands detected for all but QE10-N1 and QE10-N38 by both methods. The QE10-N1 protein preparation contained additional minor bands of ∼22 kDa and ∼29 kDa. The QE10-N38 preparation contained a minor band (∼28 kDa) about twice the size of the primary protein suggesting that it might be a protein dimer rather than a contaminant. The reason why the recombinant QE10-N14 protein with a 61 aa N-terminal truncation failed to bind the Ni^2+^-HRP probe following SDS-PAGE and transfer to a nitrocellulose membrane is not known, but possibly it was due to some refolding of the protein that rendered the N-terminal His_6_-tag inaccessible [19]. Yields of the purified proteins were estimated to be in the order of 10–30 µg/ml of bacterial culture. Moreover, the identity of each recombinant N protein was confirmed by matrix-assisted laser desorption ionization time-of-flight (MALDI-TOF) mass spectrometry analyses of peptides generated following tryptic digestion (**data not shown**).

### GAV N protein binding to synthetic nucleic acids

**Figure 2:**
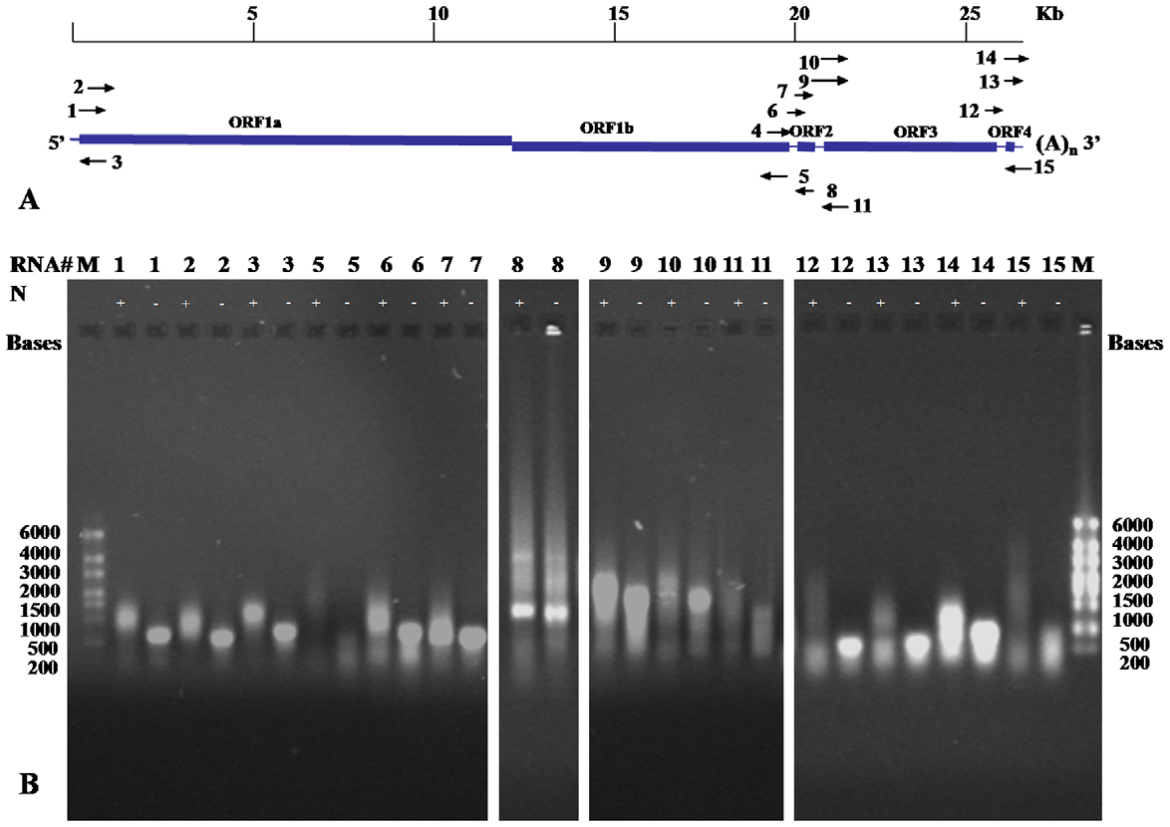
EMSAs using GAV ssRNAs incubated with full length N protein. (A) Schematic representation of the relative GAV genomic location and orientation of each of the 15 ssRNAs synthesized comprising 10 positive-sense (ssRNA-1, -2, -4, -6, -7, -9, -10, -12, -13, -14) and 5 negative-sense (ssRNA-3, -5, -8, -11, -15) RNAs. (B) Agarose gel EMSA of each ssRNA (except ssRNA-4) incubated with full-length GAV N protein. Reactions loaded on the gel contained 300 ng ssRNA incubated with (+) or without (-) 1.0 µg His_6_-N protein (RNA: protein molar ratios between 1∶17 and 1∶71) in binding buffer containing 100 mM NaCl and 50% glycerol. RNA was stained with ethidium bromide and detected using a UV transilluminator. M  =  ssRNA ladder (Ambion).

To assess the ability of the recombinant GAV N proteins to bind RNA *in vitro*, a total of 15 (+) and (−) sense ssRNAs (**Fig. 2A**) synthesized to different regions of the GAV genome (**Supplementary Table S1**) were examined in electrophoretic mobility shift assays (EMSAs). GAV genome regions selected for analysis included the 5′- and 3′-terminal sequences, intergenic regions, and sequences in ORF1a, ORF1b, ORF2, ORF3 and ORF4. Of the 15 synthetic ssRNAs, all but one (ssRNA-4) resolved as a single band following electrophoresis in a non-denaturing agarose gel.

**Figure 3:**
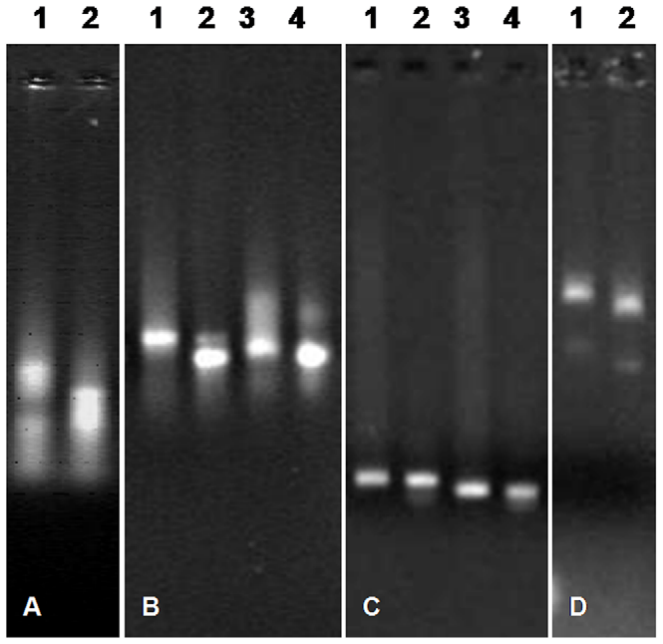
EMSAs using various RNA, and DNA sequences incubated with full-length GAV His_6_-N protein. (A) MoV M segment synthetic ssRNA incubated with (lane 1) or without (lane 2) N protein (molar ratio of RNA: N protein  = 1∶60). (B) GAV dsRNA-1/3 (lanes 1, 2) and dsRNA-6/8 (lanes 3, 4) incubated with (lanes 1, 3) and without (lanes 2, 4) N protein. (C) dsDNA-1/2 incubated with (lane 1) and without (lane 2) N protein. (D) Bacteriophage M13 ssDNA incubated with (lane 1) and without (lane 2) N protein. Reaction conditions and nucleic acid detection are as described in **Fig. 2**.

EMSA binding reactions (20 µl) were conducted at pH 8.0 in the presence of 100 mM NaCl and contained 300 ng each synthetic ssRNA and 1 µg full-length GAV His_6_-N protein (QE10-N2) equivalent to RNA:protein molar ratios ranging from 1∶17 to 1∶71 for the longest to shortest ssRNAs. Using these binding conditions, EMSAs showed that the GAV N protein could bind to all ssRNAs examined irrespective of sequence or polarity (**Fig. 2B**). Migration shifts were smaller with the shorter ssRNAs (i.e., ssRNA-12, -13, -14 and -15), with protein-bound ssRNA complexes migrating as smears slightly behind free ssRNA. The likely reason for this was the RNA:N protein molar ratios of the shorter ssRNAs (approximately 1∶17 for ssRNA-12 and -13, and 1∶27 for ssRNA-14 and -15), which were considerably lower than for the longer ssRNAs. In the presence of the full-length GAV N protein, migration of a 849 nt ssRNA synthesized to the Mourilyan virus (MoV) M RNA segment was retarded similarly to equivalent-sized GAV ssRNAs (**Fig. 3A**).

GAV N protein binding to two dsRNAs was also assessed (**Fig. 3B**). For dsRNAs prepared from (+) ssRNA-1 and (−) ssRNA-3 to the genome 5′-terminus and from (+) ssRNA-6 and (−) ssRNA-8 to the ORF2 gene, migration in the presence of the full-length N protein was retarded as evidenced by a trailing smear above the primary dsRNA band suggestive of variability in N protein binding amounts.

GAV N protein binding to dsDNA and ssDNA was also assessed (**Figs. 3C and 3D**). Using dsDNAs comprising PCR products used as templates to synthesize GAV ssRNA-1 and ssRNA-2 and the same reaction conditions used to assess ssRNA binding, no evidence was obtained for the migration of either dsDNA being retarded, although some smeared material that migrated more slowly than the primary band was observed (**Fig. 3C**). For the 6.4 kb genomic ssDNA of bacteriophage M13, migration was clearly retarded in the presence of full-length N protein (**Fig. 3D**).

**Figure 4:**
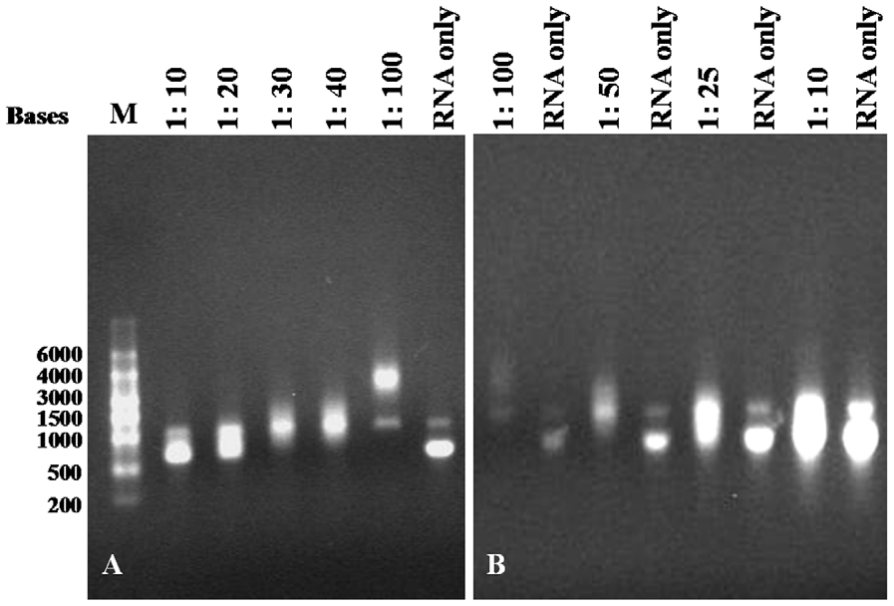
EMSAs using ssRNA-1 and full-length GAV N protein at varying molar ratios. (A) Constant ssRNA amount incubated with increasing amounts of N protein to generate ssRNA:N protein molar ratios ranging from 1∶10 to 1∶100. (B) Constant N protein amount incubated of with increasing amounts of ssRNA to generate ssRNA: N protein molar ratios ranging from 1∶100 to 1∶10. The faint bands observed are residual dsDNA not completely digested following ssRNA synthesis. Reaction conditions and nucleic acid detection are as described in **Fig. 2**.

To investigate interactions between the GAV N protein and ssRNA in more detail, EMSAs were undertaken using varied molar ratios of full-length N protein and ssRNA-1 (**Fig. 4**). Using a constant amount of ssRNA in the presence of increasing amounts of N protein (ssRNA:protein molar ratios of 1∶10 to 1∶100), ssRNA migration was clearly retarded at a ratio of 1∶20 and at ratios of 1∶30 or greater, little if any ssRNA with unaltered migration was evident (**Fig. 4A**). Starting with a ssRNA:protein ratio of 1∶100, ssRNA amounts were then increased progressively to return the ssRNA:protein molar ratio to 1∶10, and ssRNA migration was observed to be retarded less as ssRNA amounts increased (**Fig. 4B**). From either data set, it was evident the ssRNA migration was retarded to similar extents at comparable ssRNA:protein molar ratios (**Figs. 4A and 4B**).

### RNA-binding domain localized to a GAV N protein N-terminal region

**Figure 5:**
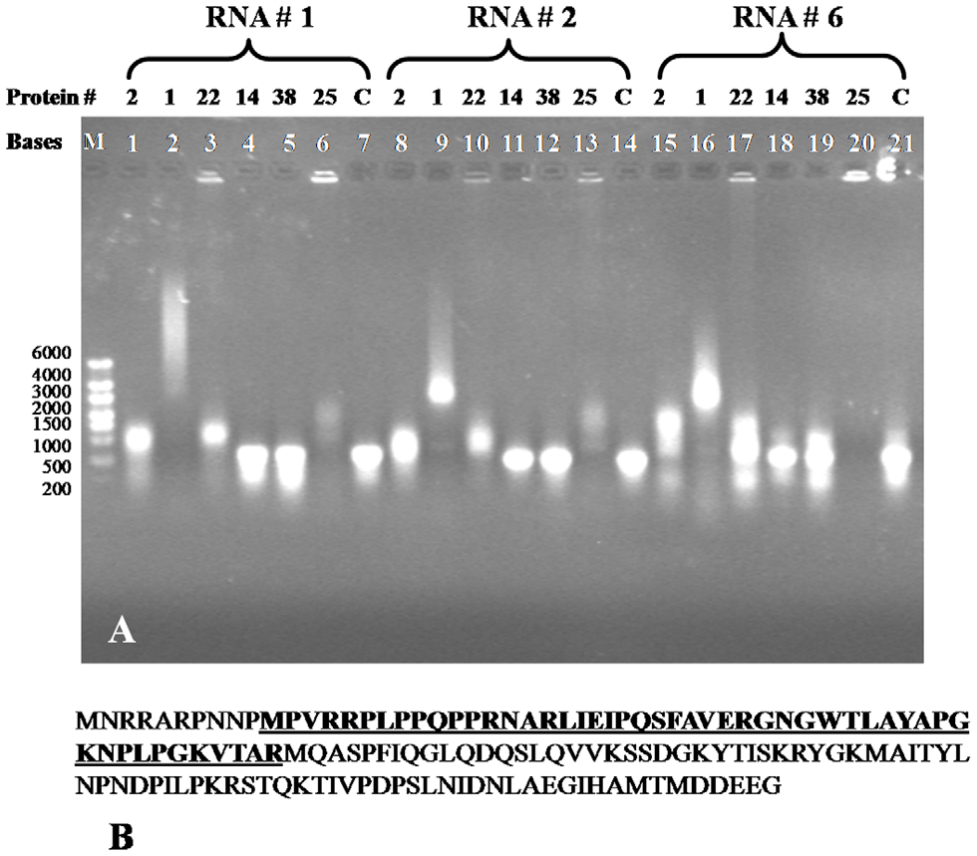
EMSAs using various synthetic GAV ssRNAs and truncated GAV N protein constructs. (A) ssRNA-1, -2 and -6 incubated with full-length N protein (QE10-N2), N proteins with N-terminal truncations (QE10-N1, -N14, -N38), a C-terminal truncation (QE10-N22) and an N- and C-terminal truncation (QE10-N25). ssRNAs incubated in the absence of N protein are indicated (C). Reaction conditions and nucleic acid detection are as described in **Fig. 2**. (B) Amino acid sequence encoded by GAV ORF2 gene with highlighted (underlined) region determined by the EMSAs to contain the N protein RNA-binding domain.

To identify the GAV N protein domain responsible for RNA binding, EMSAs were undertaken using His_6_-N protein constructs with N- or C-terminal deletions or both (**Fig. 1A** **)** and GAV ssRNA-1, -2 and -6 (**supplementary Table S3**). As shown in **Fig. 5**, the migration of all three ssRNAs was retarded in the presence of the full-length N protein and the constructs QE10-N1 and QE10-N22 containing 10 aa N-terminal and 51 aa C-terminal deletions, respectively. In the presence of N protein construct QE10-N25 containing both a 10 aa N-terminal and a 84 aa C-terminal deletion, the amounts of ssRNA-1 and -2 detected in the gel were low and ssRNA-6 failed to enter the gel altogether. However, the migration of ssRNA-1 and -2 that entered the gel was clearly retarded. Migration of none of the three ssRNAs was retarded in the presence of the N protein constructs QE10-N14 and QE10-N38 containing N-terminal truncations of 60 aa and 95 aa, respectively.

**Figure 6:**
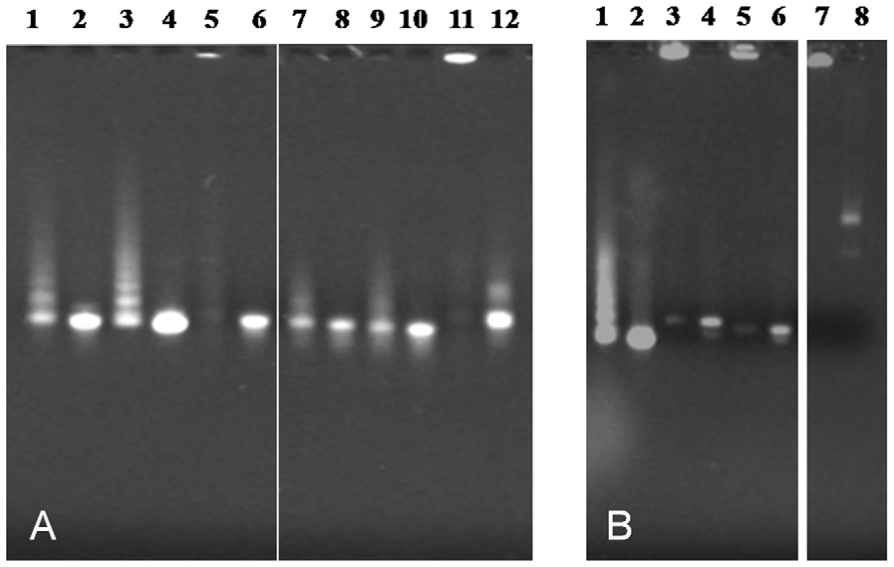
EMSAs using various synthetic GAV ssRNAs and bacteriophage M13 ssDNA incubated with a synthetic GAV N peptide. (A) EMSA using 1 µg 18 aa synthetic GAV N protein peptide and 300 ng each synthetic ssRNA to various regions of the GAV genome (ssRNA-1, -2, -3, -6, -7, -8), which based on their lengths were estimated to give ssRNA:peptide molar ratios of 1∶319, 1∶277, 1∶319, 1∶268, 1∶221 and 1∶268, respectively. The ssRNAs were incubated either with (lanes 1, 3, 5, 7, 9, 11) or without peptide (lanes 2, 4, 6, 8, 10, 12). (B) EMSA using 1 µg 18 aa synthetic GAV N protein peptide and ssRNA-2 (lane 1), dsDNA-1 and -2 (lanes 3, 5) and bacteriophage M13 ssDNA (lane 7). Reaction conditions and nucleic acid detection are as described in **Fig. 2**.

As these data indicated that the N-terminus of the GAV N protein was essential for ssRNA binding, an 18 aa synthetic peptide M^11^PVRRPLPPQPPRNARLI^29^ corresponding to a proline/arginine-rich domain in this region was tested in EMSAs for its ability to bind to various ssRNAs (**Fig. 6A**). Using ssRNA-1, -2, -3, -6, -7 and -8 at ssRNA:peptide molar ratios of approximately 1∶319, 1∶277, 1∶319, 1∶268, 1∶221 and 1∶268, respectively, the migration of each ssRNA was retarded substantially. Trailing ladder-like patterns were also evident particularly for ssRNA-1 and ssRNA-2, possibly due to the ssRNA-peptide complexes aggregating or forming multimers. For ssRNA-3 and ssRNA-8, ssRNAs were so aggregated in the presence of peptide that little ssRNA was able to enter the gel. Similarly when peptide binding to bacteriophage M13 ssDNA was assessed, ssDNA-peptide complexes or aggregates formed that precluded any ssDNA from migrating into the gel (**Fig. 6B**). The same effect was observed when dsDNA PCR products used to prepare ssRNA-1 and ssRNA-2 were incubated with the peptide, although small quantities of non-aggregated dsDNA with unaffected migration were also evident in the gel (**Fig. 6B**).

## Discussion

Here we have characterized the RNA binding properties and identified the RNA binding domain of the GAV N protein using agarose gel EMSA analysis of various synthetic RNAs reacted with various recombinant N protein constructs expressed in bacteria as well as a synthetic peptide. Purified full-length GAV N protein bound readily to synthetic (+) and (−) sense ssRNAs prepared to various GAV genome regions. The N protein also bound to a (+) sense ssRNA prepared to the M RNA segment of Mourilyan virus (MoV), a shrimp bunya-like virus, to dsRNAs prepared from (+) and (−) sense GAV ssRNAs, and to genomic ssDNA of bacteriophage M13 but not to dsDNA amplified by RT-PCR from two GAV genome regions. The ability to bind RNA was expected due to the structural role of the N protein in okavirus nucleocapsids [2], [3], [5], [20]. The fact that RNAs were bound irrespective of their sequence or polarity indicates that specific nucleation sequences, RNA folding structures or other factors might be needed to direct the GAV N protein to encapsidate genomic (+) ssRNA, as with the N proteins of many viruses, including coronaviruses [8], [21], which share the ability to bind RNA in non-sequence specific manner. This may be due to the basic charge properties of these N proteins. As the GAV N protein possesses more basic amino acids (20/144 = 13.9%, pI 9.84) than acidic amino acids (13/144 = 9.0%) [2], its net positive charge is likely to promote association with polyanions such as RNA soon after its synthesis. However, the processes directing specific binding of okavirus N protein to genomic RNA to form nucleocapsids in the potential presence of other RNAs also capable of binding clearly need to be investigated further, and experiments to examine this would be assisted greatly by shrimp cell culture systems capable supporting virus replication becoming available.

To localize the nucleic acid binding domain, recombinant His_6_-tagged GAV N proteins were expressed in *E. coli* from various plasmid constructs containing the full-length ORF2 gene or five gene fragments designed to generate variable-length N- and/or C-terminal truncations. The presence of the His_6_-tag allowed the proteins to be purified by Ni^2+^-affinity chromatography and in SDS-PAGE analyses, all six purified recombinant N proteins possessed masses equivalent to those predicted from the length of truncations, the addition of the N-terminal His_6_-sequence, and knowledge that the masses of the GAV and YHV N proteins estimated by SDS-PAGE are higher than those deduced from their amino acid sequences [3], [4], [22]. EMSA data generated using three different ssRNAs reacted with these truncated GAV N protein constructs localized the RNA binding domain to a 50 aa N-terminal sequence spanning Met^11^–Arg^60^. In this region, the GAV N protein sequence is quite basic (theoretical pI = 11.72) due to the presence of eight positively-charged residues and only two negatively-charged residues. Database searches undertaken using the InterProscan program (http://www.ebi.ac.uk/InterProScan/) [23] were unable to identify sequences close in similarity to other better characterized RNA-binding or RNA-recognition motifs. However, some general sequence similarities, mainly relating to the relatively high proportion of arginine residues, were noted with the short arginine-rich motifs (ARMs) that mediate RNA binding of the *Rev* and *Tat* proteins of human immunodeficiency virus (HIV) [24], and of the anti-terminator N protein of bacteriophages ϕ21 and P22 [25].(**Table 1**)

**Table 1:** Arginine-rich RNA-binding domains in the proteins of several viruses including the N protein of GAV and YHV.

Virus	Protein	Function	RNA-binding domain 1
HIV 1	HIV Rev	HIV (Pre-) mRNA export	TRQARRNRRRWRERQ
HIV 1	HIV Tat	Transcription trans-activator	ALGISYGRKKRRQRRRP
λ phage	l N	Transcription anti-termination	MDAQTRRRERRAEKQAQW
ϕ phage	f 21 N	Transcription anti-termination	GTAKSRYKARRAELIAER
P22 phage	P22 N	Transcription anti-termination	GNAKTRRHERRRKLAIER
GAV	GAV N	Nucleocapsid protein	MPVRRPLPPQPPRNARLI
YHV	YHV N	Nucleocapsid protein	MPRRRLPPSNRPTRNARLI

1 RNA-binding domain data taken from Burd and Dreyfuss [36].

The propensity of the Met^11^–Arg^60^ region of the GAV N protein to bind nucleic acids was confirmed in EMSA analyses of aproline-and arginine-rich peptide sequence (M^11^PVRRPLPPQPPRNARLI^29^) synthesized to the first 18 aa portion of region, and which was found to bind strongly to ssRNA as well as ssDNA and dsDNA. Proline-rich protein sequences are known to primarily form helices rather than β-sheets, and in the p33 replicase protein of tomato bushy stunt virus (TBSV), for example, an arginine/proline-rich RPRRRP motif has been shown to bind ssRNA in preference to dsRNA or dsDNA [26]. However, as the N protein peptide displayed little obvious binding preference for ssRNA compared to dsDNA, additional analyses using variant peptide sequences will be needed to identify which amino acids are responsible for conferring this broad nucleic acid binding capability.

The N protein of the coronavirus mouse hepatitis virus (MHV) has also been found to possess an ability to bind both genomic and sub-genomic viral RNAs as well as cellular RNAs to form ribonucleoprotein (RNP) complexes [27]. However, during the encapsidation process, association of the viral membrane (M) protein confers selective binding of genomic-length (+) ssRNA directed by a 190 nt packaging signal positioned toward the 3′-end of the ORF1b gene [27]. In a preliminary attempt to identify an RNA packaging signal in the GAV genome, EMSAs were performed using ssRNAs synthesized to various genome regions including (i) an ORF1b gene 3′-region spanning the relative position to the genome packaging signal identified in MHV [28], (ii) a 3′-terminal genome region corresponding in position to the region in the infectious bronchitis virus (IBV) genome reported to contain an RNA binding domain [29] and (iii) the 5′-genomic RNA terminus which, in coronaviruses, has also been reported to interact specifically with N protein [30]. However, recombinant GAV N protein bound all synthetic ssRNAs tested and whilst it is possible that its binding affinity and/or interaction kinetics varied amongst the RNAs, these could not be quantified using the EMSA method. Thus, based on this uniformity of binding, other experimental approaches will be needed to identify what sequence, if any, in the GAV genome acts as a packaging signal or confers specificity for N protein binding. As in MHV, the identification of such RNA packaging signals will likely require establishment of cell culture systems to allow the characterization of sequences of mutant defective-interfering genomic RNAs packaged into virions [28], [31].

In the EMSAs, increasing the molar ratio of N protein retarded ssRNA migration more significantly, suggesting the binding of multiple N protein molecules to each RNA and/or interactions occurring between N protein-ssRNA complexes. Based on molar ratios examined using a 626 nt ssRNA, migration was visibly retarded more when the estimated molar N protein:ssRNA ratio was increased from 20∶1 to 30∶1, and whilst no additional retardation was evident at a ratio of 50∶1, it increased again at a ratio of 100∶1. This apparent biphasic N protein ssRNA interaction suggests that there is some point at which N protein binding becomes saturated, but once present in vast excess, either more than one complex type can form or unit complexes aggregate. A similar phenomenon occurs in prunus necrotic ringspot virus, a plant ilarvirus containing a tripartite (+)ssRNA genome, in which two different complex types can form in a non-sequence-specific manner between the 32 kDa movement protein (MP) and ssRNA4 [32]. One ssRNA complex forming in the presence of high MP amounts (MP:ssRNA molar ratio 400∶1) was observed to enter an EMSA gel, whereas another complex type that formed at a lower molar ratio (120∶1) did not. Moreover, urea denaturation had little in any effect on the type of ssRNA:MP complex formed at higher MP amounts but disaggregated the complex type formed in lower MP amounts, suggested that the latter might comprise rod-like structures restricting gel entry and that excess MP might promote increased protein-protein interactions resulted in a more compact globular ssRNA-MP complex capable of entering the gel matrix [32]. Although something similar might be the reason for the biphasic interaction of the GAV N protein with ssRNA, delineation of the nature of the various complexes that can form will require further investigation.

Based on its structural role in nucleocapsids [4], [5], it was expected that the GAV N protein would be capable of binding ssRNA. Indeed such ssRNA binding activity was confirmed in EMSA analyses of various RNAs and recombinant N protein constructs. Moreover, analyses with variably truncated N protein constructs as well as a synthetic peptide showed that this activity was not dictated by any specific sequence constraints and was localized to a short, highly-charged N-terminal motif rich in arginines and prolines. Whilst these findings advance our understanding of the RNA binding capabilities of the N protein of the crustacean okaviruses, additional studies are now needed to determine the mechanism by which genomic ssRNA is nucleated either specifically or preferentially.

## Materials and Methods

### Cloning and expression of full-length and truncated GAV His_6_-tagged N proteins

The GAV ORF2 gene and sequences with variable 5′- and/or 3′-terminal truncations (**Fig. 1A**) were amplified by PCR using various primers containing *Bam* HI sites (**Supplementary Table S2**) to allow in-frame insertion with the N-terminal His_6_-tag of pQE10 (QIAGEN). A plasmid containing the entire ORF2 gene [2] was used as template for all PCRs. Amplified DNA products were digested with *Bam* HI and ligated into pQE10. Plasmid DNA was transformed into competent DH5α or M13[pREP4] *E. coli* host strains and clones were grown on Luria broth (LB) agar plates containing 100 µg/ml ampicillin or 100 µg/ml ampicillin plus 25 µg/ml kanamycin. To screen clones for ORF2 inserts in the correct orientation, rapid colony PCRs were performed using the pQE-specific primer 5′-CCCGAAAAGTGCCACCTG-3′ (QIAGEN) in combination with various ORF2 gene-specific anti-sense primers (**Supplementary Table S2**). Plasmid DNA prepared to each clone selected for analysis was sequenced using the pQE-specific primer and clone specific reverse primers to confirm the fidelity of the DNA insert.

Two plasmid clones containing the inserts were each inoculated into 2 ml LB medium containing 100 µg/ml ampicillin (plus 25 µg/ml kanamycin for M13[pREP4] cells) and grown overnight at 37°C in a shaking incubator. A portion (500 µl) of each culture was inoculated into 10 ml pre-warmed LB medium containing antibiotics and the culture was shaken vigorously at 37°C until the optical density (OD) at 600 nm had reached 0.5. Protein expression was induced by the addition of 0.1 to 1.0 mM final concentration of isopropyl-β-D-1-thiogalactopyranoside (IPTG) and incubation at 37°C was continued overnight. Cells were collected by centrifugation (8,000×*g*, 10 min, 4°C), disrupted by sonication in lysis buffer (300 mM NaCl, 10 mM imidazole, 50 mM NaH_2_PO_4_ pH 8.0) for non-denatured protein purification and the ORF2 His_6_-fusion proteins were purified using Ni^+2^-NTA agarose beads as described in the QIAexpressionist^TM^ instruction manual (QIAGEN). The proteins were stored at −80°C until used in EMSAs. For some ORF2 His_6_-fusion proteins, *E. coli* cells were lysed in 8 M urea and protein was purified under denaturing conditions as described in the QIAexpressionist^TM^ manual. Purified denatured proteins (500 µl each) were refolded by dialysis overnight at 4°C in 200 ml refolding buffer (3 M urea, 500 mM NaCl, 10 mM reduced glutathione, 1 mM oxidized glutathione, 1 mM EDTA, 20 mM Tris-HCl pH 7.1). The dialysis buffer was then replaced with 200 ml PBS and dialysis was continued for 24 h at 4°C.

### Quantification of recombinant proteins

Purified His_6_-ORF2 fusion proteins were concentrated by centrifugation using either a YM3 or YM10 Centricon^TM^ membrane concentrator (Millipore) and protein yields were quantified using a BCA protein assay kit (Pierce) and also estimated visually in comparison to protein standards resolved by SDS-PAGE and stained with Coomassie brilliant blue (CBB) R250. Yields of purified proteins were in the order of ∼4 mg each.

### SDS-PAGE separation of proteins and Western blotting

An aliquot of each purified recombinant His_6_-ORF2 protein was separated by SDS-PAGE using a 15% polyacrylamide gel run at 120 V for 90 min. A pair of identically loaded gels was separated simultaneously. Proteins in one gel were stained with CBB **(** **Fig 1B** **)** and proteins in the other were electro-transferred onto a Hybond^TM^-C nitrocellulose membrane (Amersham) using Towbin transfer buffer (25 mM Tris, 192 mM glycine, 20% methanol and 0.1% SDS) and a Hoefer semi-dry protein transfer system run at 225 mA for 90 min. The membrane was blocked with 5% skim milk powder in Tris-buffered saline (TBS; 0.9% NaCl in 100 mM Tris-HCl pH 7.5) for 15 min. The blocked membrane was washed with three changes of TBS for 10 min each before being incubated for 35 min in Ni^2+^-HRP (Sigma-Aldrich) diluted 1∶1,000 in TBS. The membrane was washed with three changes of TBS for 10 min each and incubated in developing solution (30 mg 4-chloronaphtol, 2.5 ml ice-cold methanol and 20 µl hydrogen peroxide in TBS, 50 ml) for 30 s to 3 min to detect color signal. Once a positive signal was observed, the reaction was stopped by incubation in TBS for 10 min. Images on membranes **(** **Fig. 1C** **)** and CBB-stained gels were captured using an EPSON Perfection V200 photograph scanner.

### Synthetic peptide

The synthetic peptide (MPVRRPLPPQPPRNARLI) was purchased from Shanghai Science Peptide Biological Technology Co. Ltd., China. The lyophilized peptide, reported by the manufacturer to be 99.2% pure as determined by high performance liquid chromatography (HPLC), was dissolved to appropriate concentration in double-distilled water.

### Preparation of ssRNA

Plus- and minus-sense ssRNAs were synthesized from PCR products amplified from plasmids containing cDNA inserts corresponding to various regions of the GAV genome **(** **Fig. 2A** **)**. The plasmids included: (i) pGAV22, containing a 623 nt ORF1a gene sequence extending to the 5′-terminus of the genome; (ii) pGAV16, containing a 4282 nt sequence beginning 509 nt upstream of the ORF1b gene 3′-terminus and extending to a position 3773 nt downstream of the ORF3 gene 5′-terminus; and (iii) pGAV12.1, containing a ∼2.7 kb insert extending from a region within the ORF3 gene to the GAV genome 3′-polyA tail **(Supplementary Table S1)**. Sequences of PCR primers used to amplify DNA products are shown in **Supplementary Table S3**. In each PCR, either the sense or the anti-sense GAV-specific primer included a 5′-terminal T7 RNA polymerase promoter sequence. Each PCR contained ∼100 ng plasmid DNA, 25 pmol of each primer, 5 µl 10× PCR buffer (670 mM Tris-HCl pH 8.8), 3 µl 25 mM MgCl_2_, 1 µl 10 mM dNTP mix, 0.5 µl 5.5 U/µl *Taq* DNA polymerase (Promega) and DNase/RNase-free water was added to final volume of 50 l. Thermal cycling conditions used in the PCRs were 95°C for 4 min, 35 cycles of 95°C for 30 s, 56°C for 30 s, and 72°C for 120 min, followed by 72°C for 7 min. PCR products were purified using a QIAquick^TM^ gel purification kit (QIAGEN) according to the manufacturer's instructions.

Purified DNA products were treated with Klenow DNA polymerase to remove 3′-adenosine overhangs and end-filled using *Taq* DNA polymerase prior to RNA synthesis. To synthesize RNA, 1 µg purified DNA was added to a 20 µl reaction prepared using the MEGAscript^TM^ high yield *in vitro* RNA transcription kit (Ambion), and RNA synthesis and DNA removal were performed according to the kit instructions. Synthetic RNA was purified using a MEGAclear^TM^ RNA purification column (Ambion), eluted in 100 µl elution buffer (10 mM Tris-HCl pH 8.0) and stored at −80°C. Mourilyan virus (MoV) RNA was synthesized using 1 µg *Pst* I-linearized pMoV4.1 DNA containing a 849 nt cDNA corresponding to the M (membrane glycoproteins G1/G2) ssRNA genome segment [33], [34] as a template for *in vitro* T7 RNA transcription.

### Preparation of dsRNA

Double-stranded (ds)RNAs corresponding to the GAV genome 5′-terminus and a sequence including the intergenic region downstream of ORF1b to the end of ORF2 were prepared by adding together equimolar amounts of plus- and minus-sense ssRNAs in MEGAclear^TM^ (Ambion) elution buffer, heating at 95°C for 10 min and then annealing at room temperature for 30 min. The formation of dsRNA was confirmed by comparing its migration to each ssRNA in a 1% agarose-TAE (40 mM Tris-acetate pH 8.0, 1 mM EDTA) gel.

### Preparation of dsDNA and ssDNA

Three PCR products selected randomly were used to assess N-protein binding to dsDNA in EMSAs. Single-stranded DNA extracted from bacteriophage M13 using phenol chloroform extraction was kindly provided by Dr Andrew McDevitt, CSIRO Livestock Industries, Australia.

### Electrophoretic mobility shift assay

An electrophoretic mobility shift assay (EMSA) was used to identify N-protein binding to nucleic acid [32], [35]. In each assay, 300 ng synthetic ssRNA/ssDNA/dsDNA in MEGAclear^TM^ elution buffer (Ambion) was heated at 85°C for 5 min and cooled at room temperature for 15 min before addition of GAV His_6_-N fusion protein or synthetic peptide (1 µg each), 10 µl binding buffer (100 mM NaCl, 50% glycerol in 10 mM Tris-HCl pH 8.0) and 2 units of RiboLock^TM^ RNase inhibitor (Fermentas). The reaction volume was adjusted to 20 µl with diethyl pyrocarbonate (DEPC)-treated water and it was incubated at room temperature for 30 min. Following the addition of 2 µl 1% bromophenol blue tracking dye, the RNA-protein sample was separated by electrophoresis in a 1% agarose-TAE gel containing 0.5 µg/ml ethidium bromide. The relative migration of free nucleic acid and nucleic acid-N protein complexes was visualized using a UV transilluminator and gel images were recorded using a Gel-Doc documentation system (BioRad). The molar ratio of ssRNA-1 to full-length His_6_-N protein (pQE10-N2) in the reaction was 1∶42. To study the effect of relative RNA and N protein concentrations on binding, varying molar ratios of RNA:N protein (1∶0, 1∶10, 1∶20, 1∶25, 1∶30, 1∶50, 1∶100) were also tested.

## Supporting Information

Table S1 Details of each GAV synthetic ssRNA examined including positions in GAV genome, polarities and length and the plasmid DNA used as template for PCR.
(DOC)

Table S2 PCR primers used to amplify full-length and truncated ORF2 gene coding sequences to construct pQE10 GAV N protein expression vectors and also used in colony PCRs to determine insert orientations following cloning.
(DOC)

Table S3 PCR primers used to amplify DNA templates for ssRNA synthesis using T7 RNA polymerase.
(DOC)

## References

[1] Gorbalenya AE, Enjuanes L, Ziebuhr J, Snijder EJ (2006). Nidovirales: Evolving the largest RNA virus genome.. Virus Research.

[2] Cowley JA, Cadogan LC, Spann KA, Sittidilokratna N, Walker PJ (2004). The gene encoding the nucleocapsid protein of Gill-associated nidovirus of *Penaeus monodon* prawns is located upstream of the glycoprotein gene.. Journal of Virology.

[3] Sittidilokratna N, Phetchampai N, Boonsaeng V, Walker PJ (2006). Structural and antigenic analysis of the yellow head virus nucleocapsid protein p20.. Virus Research.

[4] Cowley JA, Cadogan LC, Wongteerasupaya C, Hodgson RAJ, Boonsaeng V (2004). Multiplex RT-nested PCR differentiation of gill-associated virus (Australia) from yellow head virus (Thailand) of *Penaeus monodon*.. Journal of Virological Methods.

[5] Soowannayan C, Flegel TW, Sithigorngul P, Slater J, Hyatt A (2003). Detection and differentiation of yellow head complex viruses using monoclonal antibodies.. Diseases of Aquatic Organisms.

[6] Wijegoonawardane PKM, Cowley JA, Phan T, Hodgson RAJ, Nielsen L (2008). Genetic diversity in the yellow head nidovirus complex.. Virology.

[7] Sithigorngul P, Rukpratanporn S, Longyant S, Chaivisuthangkura P, Sithigorngul W (2002). Monoclonal antibodies specific to yellow-head virus (YHV) of Penaeus monodon.. Diseases of Aquatic Organisms.

[8] Masters PS, Sturman LS (1990). Background paper. Functions of the coronavirus nucleocapsid protein.. Advances in Experimental Medicine and Biology.

[9] Snijder EJ, den Boon JA, Spaan WJ, Verjans GM, Horzinek MC (1989). Identification and primary structure of the gene encoding the Berne virus nucleocapsid protein.. Journal of General Virology.

[10] Kroneman A, Cornelissen LAHM, Horzinek MC, de Groot RJ, Egberink HF (1998). Identification and characterization of a porcine Torovirus.. Journal of Virology.

[11] Wootton SK, Nelson EA, Yoo D (1998). Antigenic structure of the nucleocapsid protein of porcine reproductive and respiratory syndrome virus.. Clinical and Diagnostic Laboratory Immunology.

[12] Duckmanton LM, Tellier R, Liu P, Petric M (1998). Bovine torovirus: Sequencing of the structural genes and expression of the nucleocapsid protein of Breda virus.. Virus Research.

[13] Lapps W, Hogue BG, Brian DA (1987). Sequence analysis of the bovine coronavirus nucleocapsid and matrix protein genes.. Virology.

[14] Williams AK, Li W, Sneed LW, Collisson EW (1992). Comparative analyses of the nucleocapsid genes of several strains of infectious bronchitis virus and other coronaviruses.. Virus Research.

[15] Liu P, Yang J, Wu X, Fu ZF (2004). Interactions amongst rabies virus nucleoprotein, phosphoprotein and genomic RNA in virus-infected and transfected cells.. Journal of General Virology.

[16] Mavrakis M, Mehouas S, Real E, Iseni F, Blondel D (2006). Rabies virus chaperone: Identification of the phosphoprotein peptide that keeps nucleoprotein soluble and free from non-specific RNA.. Virology.

[17] Masters PS (1992). Localization of an RNA-binding domain in the nucleocapsid protein of the coronavirus mouse hepatitis virus.. Archives of Virology.

[18] Zuniga S, Sola I, Moreno JL, Sabella P, Plana-Duran J (2007). Coronavirus nucleocapsid protein is an RNA chaperone.. Virology.

[19] Lesley SA, Graziano J, Cho CY, Knuth MW, Klock HE (2002). Gene expression response to misfolded protein as a screen for soluble recombinant protein.. Protein Engineering.

[20] Cowley JA, Dimmock CM, Spann KM, Walker PJ (2001). Gill-associated virus of *Penaeus monodon* prawns - Molecular evidencefor the first invertebrate nidovirus.. Nidoviruses (Coronaviruses and Arteriviruses).

[21] Youn S (2003). In vitro assembly of an infectious cDNA clone of infectious bronchitis virus and its application as a gene transfer vector..

[22] Nadala ECB, Tapay LM, Loh PC (1997). Yellow-head virus: a rhabdovirus-like pathogen of penaeid shrimp.. Diseases of Aquatic Organisms.

[23] Zdobnov EM, Apweiler R (2001). InterProScan - an integration platform for the signature-recognition methods in InterPro.. Bioinformatics.

[24] Tan RY, Frankel AD (1995). Structural variety of arginine-rich RNA-binding peptides.. Proceedings of the National Academy of Sciences of the United States of America.

[25] Burd CG, Dreyfuss G (1994). RNA binding specificity of hnRNP A1: significance of hnRNP A1 high-affinity binding sites in pre-mRNA splicing.. Embo Journal.

[26] Rajendran KS, Nagy PD (2003). Characterization of the RNA-binding domains in the replicase proteins of tomato bushy stunt virus.. Journal of Virology.

[27] Narayanan K, Makino S (2001). Cooperation of an RNA packaging signal and a viral envelope protein in coronavirus RNA packaging.. Journal of Virology.

[28] Makino S, Yokomori K, Lai MMC (1990). Analysis of efficiently packaged defective interfering RNAs of murine coronavirus - localization of a possible RNA-packaging signal.. Journal of Virology.

[29] Zhou M, Williams AK, Chung SI, Wang L, Collisson EW (1996). The infectious bronchitis virus nucleocapsid protein binds RNA sequences in the 3′ terminus of the genome.. Virology.

[30] Stohlman SA, Baric RS, Nelson GN, Soe LH, Welter LM (1988). Specific interaction between coronavirus leader RNA and nucleocapsid protein.. Journal of Virology.

[31] Fosmire JA, Hwang K, Makino S (1992). Identification and characterization of a coronavirus packaging signal.. Journal of Virology.

[32] Herranz MC, Pallas V (2004). RNA-binding properties and mapping of the RNA-binding domain from the movement protein of Prunus necrotic ringspot virus.. Journal of General Virology.

[33] Cowley JA, McCulloch RJ, Rajendran KV, Cadogan LC, Spann KM (2005). RT-nested PCR detection of Mourilyan virus in Australian *Penaeus monodon* and its tissue distribution in healthy and moribund prawns.. Diseases of Aquatic Organisms.

[34] Cowley JA, McCulloch RJ, Spann KM, Cadogan LC, Walker PJ, Walker PJ, Lester RG, Bondad-Reantaso MG (2005). Preliminary molecular and biological characterisation of Mourilyan virus (MoV): a new bunya-related virus of Penaeid prawns..

[35] Carey J, Sauer RT (1991). Gel retardation.. Methods in Enzymology.

[36] Burd CG, Dreyfuss G (1994). Conserved structures and diversity of functions of RNA-binding proteins.. Science.

