# Supplementary material for: RNA-Binding Domain in the Nucleocapsid Protein of Gill-Associated Nidovirus of Penaeid Shrimp
Source: PLoS One. 2011 Aug 3;6(8):e22156. doi: 10.1371/journal.pone.0022156 (PMC3153931; doi:10.1371/journal.pone.0022156)
Supplement: Table S1 — Details of each GAV synthetic ssRNA examined including positions in GAV genome, polarities and length and the plasmid DNA used as template for PCR. (DOC) [file pone.0022156.s001.doc]

Supplementary Tables

**Supplementary Table 1.** Details of each GAV synthetic ssRNA examined including positions in GAV genome, polarities and length and the plasmid DNA used as template for PCR

| **ssRNA** | **Plasmid clone**^1^ | **GAV gene(s)** | **Polarity** | **Length (nt)** | **Genome region spanned^2^** |
| --- | --- | --- | --- | --- | --- |
| 1 | 18 | 5’UTR-ORF1a | + | 626 | 1-623 |
| 2 | 18 | ORF1a | + | 558 | 69-627 |
| 3 | 18 | 5’UTR-ORF1a | - | 626 | 1-623 |
| 4 | 16 | ORF1b | + | 492 | 19,600-20,089 |
| 5 | 16 | ORF1b | - | 492 | 19,600-20,089 |
| 6 | 16 | IGR-ORF2 | + | 531 | 20,090-20,620 |
| 7 | 16 | ORF2 | + | 438 | 20,185-20,620 |
| 8 | 16 | IGR-ORF2 | - | 531 | 20,090-20,620 |
| 9 | 9 | IGR-ORF3 | + | 1060 | 20,621-21,681 |
| 10 | 9 | ORF3 | + | 1003 | 20,741-21,741 |
| 11 | 9 | IGR-ORF3 | - | 1060 | 20,621-21,681 |
| 12 | 12.1 | IGR-ORF4 | + | 261 | 25,600-25,861 |
| 13 | 12.1 | ORF4 | + | 255 | 25,861-26,116 |
| 14 | 12.1 | ORF4-3’UTR | + | 399 | 25,836-26,260 |
| 15 | 12.1 | ORF4-3’UTR | - | 399 | 25,836-26,235 |

**^1^**Plasmid clone number used as template for PCR to generate DNA products used to synthesis ssRNA

^2^Nucleotide numbers correspond to the published full-length GAV genome sequence (GenBank AY039647, AF227196, AF102827, AF102828, AF126718)

UTR = untranslated region, IGR = intergenic region
