# Supplementary material for: RNA-Binding Domain in the Nucleocapsid Protein of Gill-Associated Nidovirus of Penaeid Shrimp
Source: PLoS One. 2011 Aug 3;6(8):e22156. doi: 10.1371/journal.pone.0022156 (PMC3153931; doi:10.1371/journal.pone.0022156)
Supplement: Table S2 — PCR primers used to amplify full-length and truncated ORF2 gene coding sequences to construct pQE10 GAV N protein expression vectors and also used in colony PCRs to determine insert orientations following cloning. (DOC) [file pone.0022156.s002.doc]

**Supplementary Table 2.** PCR primers used to amplify full-length and truncated ORF2 gene coding sequences to construct pQE10 GAV N protein expression vectors and also used in colony PCRs to determine insert orientations following cloning

| No. | Name | Sequence | Pair | Length (aa) | Protein # |
| --- | --- | --- | --- | --- | --- |
| 1 | GAV269R | 5’CGGATCCTTATTTGCCGTACCTCTTTGAGAT3’ | 1 + 4 | 93 | 22 |
| 2 | GAV270R | 5’CGGATCCTTAACGAGCGGTGACCTTACCCGG3’ | 2 + 5 | 50 | 25 |
| 3 | GAV116R | 5’aattggatccttagggttgagtgtcaccttc3’ |  |  |  |
| 4 | GAV117F | 5’ggctaggatcctgac atgaaccgccgcgca3’ | 3 + 4 | 144 | 2 |
| 5 | GAV193F | 5’ctaaggatcccatgcccgtccggcgccct3’ | 3 + 5 | 134 | 1.3 |
| 6 | GAV194F | 5’tcacggatcctatgcaagcatcaccattcatt3’ | 3 + 6 | 57 | 14 |
| 7 | GAV195F | 5’ggtaggatccaatggctatcacctatcttaac3’ | 3 + 7 | 51 | 38 |

*Bam* HI sites used for in-frame insertion of PCR products into pQE10 (QIAGEN) are underlined.
