# Supplementary material for: RNA-Binding Domain in the Nucleocapsid Protein of Gill-Associated Nidovirus of Penaeid Shrimp
Source: PLoS One. 2011 Aug 3;6(8):e22156. doi: 10.1371/journal.pone.0022156 (PMC3153931; doi:10.1371/journal.pone.0022156)
Supplement: Table S3 — PCR primers used to amplify DNA templates for ssRNA synthesis using T7 RNA polymerase. (DOC) [file pone.0022156.s003.doc]

**Supplementary Table 3.** PCR primers used to amplify DNA templates for ssRNA synthesis using T7 RNA polymerase

| **RNA No.** | **Primer** | **Sequence** |
| --- | --- | --- |
| 1 | ORF1a F1 | 5’TAATACGACTCACTATAGGGacgttacgttccacgtact3’ |
|  | ORF1a R1 | 5’ tgaagaccgaacagagctct3’ |
| 2 | ORF1a F2 | 5’TAATACGACTCACTATAGGGatggagccttttgaggttc3’ |
|  | ORF1a R1 | 5’ tgaagaccgaacagagctct3’ |
| 3 | ORF1a F1 neg | 5’acgttacgttccacgtact3’ |
|  | ORF1a R1 neg | 5’ taatacgactcactatagggtgaagaccgaacagagctct3’ |
| 4 | ORF1b F1 | 5’TAATACGACTCACTATAGGGggtgagtgcccattcattc3’ |
|  | ORF1b R1 | 5’ttaaaatttgatgaatctggga3’ |
| 5 | ORF1b F1 neg | 5’ggtgagtgcccattcattc3’ |
|  | ORF1b R1 neg | 5’taatacgactcactatagggttaaaatttgatgaatctggga 3’ |
| 6 | ORF2 F1 | 5’TAATACGACTCACTATAGGGacctgatttaccacaa3’ |
|  | ORF2 R1 | 5’ttagggttgagtgtcacct3’ |
| 7 | ORF2 F2 | 5’TAATACGACTCACTATAGGGatgaaccgccgcgcac3’ |
|  | ORF2 R1 | 5’ttagggttgagtgtcacct3’ |
| 8 | ORF2 F1 neg | 5’acctgatttaccacaa3’ |
|  | ORF2 R1 neg | 5’taatacgactcactatagggttagggttgagtgtcacct3’ |
| 9 | ORF2 F3 | 5’TAATACGACTCACTATAGGGctctccaattaattggtcaat3’ |
|  | ORF3 R1 | 5’ tggtggatctaactgagattg3’ |
| 10 | ORF3 F1 | 5’TAATACGACTCACTATAGGGatgcaatgttcgcgatcatc3’ |
|  | ORF3 R1 | 5’ tggtggatctaactgagattg3’ |
| 11 | ORF3 F1 neg | 5’atgcaatgttcgcgatcatc3’ |
|  | ORF3 R1 neg | 5’taatacgactcactatagggtggtggatctaactgagattg3’ |
| 12 | ORF3 F2 | 5’TAATACGACTCACTATAGGGaccattgaattttgacatatc3’ |
|  | ORF3 R2 | 5’ aaccttaatcgggtagaataa3’ |
| 13 | ORF4 F1 | 5’TAATACGACTCACTATAGGGatgattcaaatcccgaatataa3’ |
|  | ORF4 R1 | 5’ ttagttaggggccaatgaat3’ |
| 14 | ORF4 F2 | 5’TAATACGACTCACTATAGGGgggtgatttgcatcatatca3’ |
|  | ORF4 R2 | 5’ ttttttttttttttcatatcaccg3’ |
| 15 | ORF4 F1 neg | 5’ atgattcaaatcccgaatataa3’ |
|  | ORF4 R2 neg | 5’taatacgactcactatagggttttttttttttttttcatatcaccg3’ |

T7 promoter sequences in the PCR primers are underlined
